# Gene Expression of Corals in Response to Macroalgal Competitors

**DOI:** 10.1371/journal.pone.0114525

**Published:** 2014-12-12

**Authors:** Tonya L. Shearer, Terry W. Snell, Mark E. Hay

**Affiliations:** Georgia Institute of Technology, School of Biology, 310 Ferst Dr., Atlanta, GA, 30332, United States of America; University of New South Wales, Australia

## Abstract

As corals decline and macroalgae proliferate on coral reefs, coral-macroalgal competition becomes more frequent and ecologically important. Whether corals are damaged by these interactions depends on susceptibility of the coral and traits of macroalgal competitors. Investigating changes in gene expression of corals and their intracellular symbiotic algae, *Symbiodinium,* in response to contact with different macroalgae provides insight into the biological processes and cellular pathways affected by competition with macroalgae. We evaluated the gene expression profiles of coral and *Symbiodinium* genes from two confamilial corals, *Acropora millepora* and *Montipora digitata*, after 6 h and 48 h of contact with four common macroalgae that differ in their allelopathic potency to corals. Contacts with macroalgae affected different biological pathways in the more susceptible (*A. millepora*) versus the more resistant (*M. digitata*) coral. Genes of coral hosts and of their associated *Symbiodinium* also responded in species-specific and time-specific ways to each macroalga. Changes in number and expression intensity of affected genes were greater after 6 h compared to 48 h of contact and were greater following contact with *Chlorodesmis fastigiata* and *Amphiroa crassa* than following contact with *Galaxaura filamentosa* or *Turbinaria conoides*. We documented a divergence in transcriptional responses between two confamilial corals and their associated *Symbiodinium*, as well as a diversity of dynamic responses within each coral species with respect to the species of macroalgal competitor and the duration of exposure to that competitor. These responses included early initiation of immune processes by *Montipora*, which is more resistant to damage after long-term macroalgal contact. Activation of the immune response by corals that better resist algal competition is consistent with the hypothesis that some macroalgal effects on corals may be mediated by microbial pathogens.

## Introduction

Coral reefs are in global decline. In recent decades coral cover has declined by 80% in the Caribbean and 50% throughout the tropical Pacific [1], [2]. As corals decline and are replaced by macroalgae, coral-macroalgal interactions increase for the remaining corals [3]. This can result in further coral damage and decline through both impacts on established corals [4]–[11] and through suppressing the recruitment and survival of larval and juvenile corals [12]–[19]. This further accelerates reef degradation, destabilizing the function of coral reef ecosystems [20], .

The diversity of biological, morphological, and chemical properties of macroalgae provides a range of potential mechanisms by which seaweeds may impact corals [6], including shading, abrasion, overgrowth, allelopathy, disease transmission, and alteration of coral-associated microbial mutualists. These impacts range from no visual impact to moderate or severe bleaching with partial or complete colony mortality [3], [10], [11], [22]–[25]. In addition to the variety of mechanisms by which macroalgae may damage corals, variation in the physiological resilience of different coral species increases the complexity of these interactions. While some corals are strongly impacted by contact with macroalgae, others are more resistant to damage by these same competitors [3], [10]. For example, *Montipora digitata* was less susceptible to damage by macroalgal allelopathy, as quantified by measurements of bleaching, mortality and reductions in photosynthetic activity of *Symbiodinium*, relative to *Acropora millepora* and *Pocillopora damicornis* [10].

Gene expression analyses provide insight into pathways affected by stressors and into potential mechanisms affecting coral survivorship [26]–[28]. Although changes in gene expression do not necessarily translate to alterations in protein abundance and effects on fitness [29], transcriptional changes in specific genes can be indicative of physiological responses to environmental stressors. For example, population-specific resistance to thermal stress in *Acropora hyacinthus* was attributed to a higher constitutive level of expression of stress-related genes in tolerant corals [27], enhancing survivorship during periods of high water temperatures.

Understanding how corals respond to environmental stressors, including contact with macroalgae, is complicated due to symbiotic association with intracellular photosynthetic dinoflagellates (*Symbiodinium* spp.) and other symbionts associated with corals. Tolerances and stress responses of *Symbiodinium* may influence overall coral health [30], [31]. Because macroalgal contact is growing in importance as a coral stressor, it is important to understand how the holobiont (the coral host and its associated symbionts) is affected by competition with macroalgae. Alterations in coral and *Symbiodinium* gene expression patterns following contact with macroalgae may provide insights into how macroalgae affect holobiont health and function, but such studies may be more descriptive and comparative at present than is desirable because the function of many genes are incompletely known.

In this study, gene expression profiles of two confamilial corals, *Acropora millepora* and *Montipora digitata* (family Acroporidae), and their associated *Symbiodinium* were characterized to determine the transcriptional response after contact with four species of macroalgae (*Amphiroa crassa*, *Chlorodesmis fastigiata*, *Galaxaura filamentosa* and *Turbinaria conoides*), with which both corals commonly co-occur on fished reef flats in Fiji [3]. Given the phylogenetic relationship between these coral species and the general nature of the conserved stress response [32], one might expect similar stress response pathways to be activated due to macroalgal contact. Previous studies, however, indicate that these coral species vary in susceptibility to damage by these macroalgae after long-term exposure (20 days) [10], and the genetic basis for these differences is associated with patterns of differential gene expression [26]. To our knowledge, this is the first study to characterize and contrast gene expression responses of multiple coral species and their associated *Symbiodinium* in response to stressors over time.

## Results

**Figure 1:**
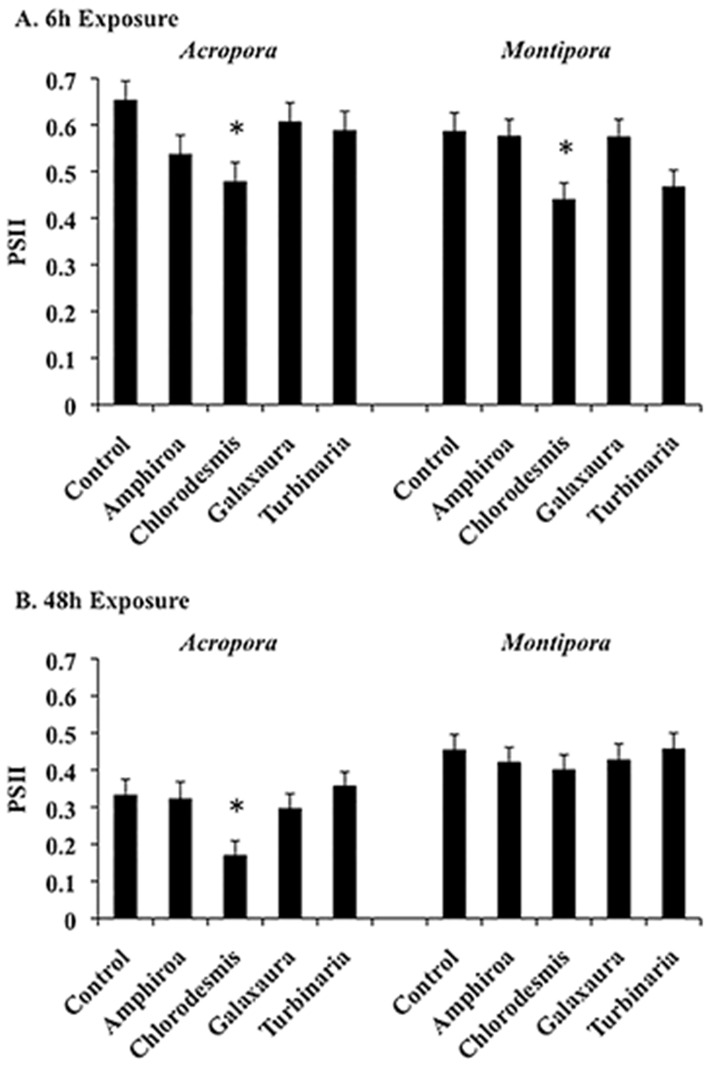
Quantification of photosystem II (PSII) effective quantum yield of *Symbiodinium* (mean ±SE) in *Acropora millepora* and *Montipora digitata* coral after 6 h (A) or 48 h (B) exposure to macroalgal tissue. N = 8–10 coral fragments per treatment. Significance was determined by ANOVA and treatments were compared to respective controls using Dunnett's post-hoc test. * indicates *P*<0.05.

Impacts of macroalgal contact on *Symbiodinium* photosynthetic activity was measured as effective quantum yield of photosystem II (Φ_PSII_). After 6 h of exposure to *Chlorodesmis fastigiata, Symbiodinium* in both *Acropora millepora* and *Montipora digitata* experienced significant ∼25% reductions in photosynthetic activity relative to controls (*P* = 0.017 and 0.032, respectively; Fig. 1A). After 48 h of exposure to *C. fastigiata*, photosynthetic activity was reduced by a significant ∼50% in *A. millepora* (*P* = 0.030; Fig. 1B), but impacts on *M. digitata* were no longer significant. The three other species of macroalgae had no significant effect on effective quantum yield of photosystem II (Φ_PSII_) after 6 h or 48 h exposures. Because *Symbiodinium* cells could not be counted in the same coral tissues used for gene expression analyses, we do not know whether the reductions in photosynthetic activity resulted from damage to the photosynthetic machinery or from reduced densities of *Symbiodinium.*

**Figure 2:**
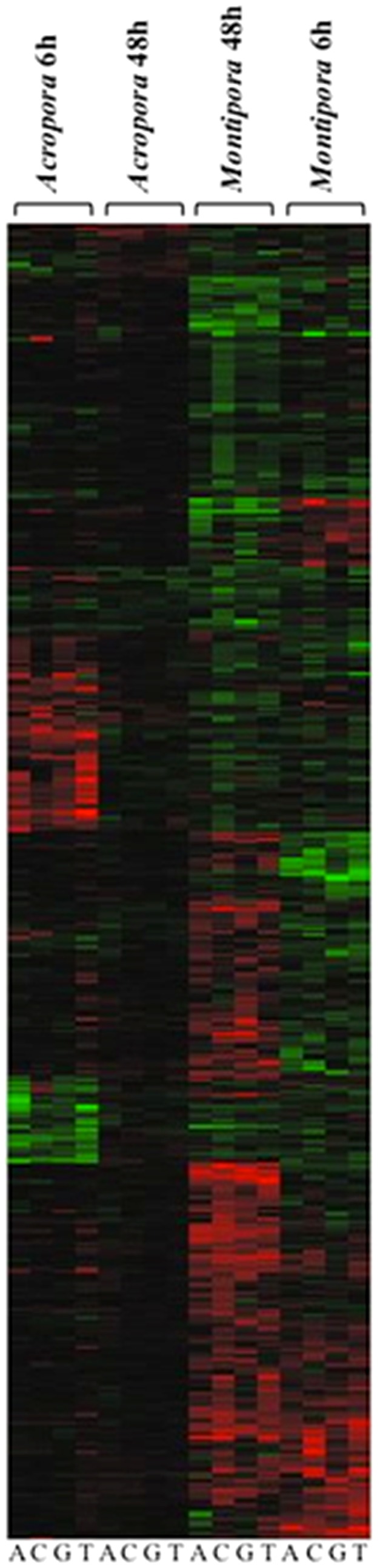
Hierarchical cluster analysis of 850 *Acropora millepora* and *Montipora digitata* genes (both coral and *Symbiodinium*) differentially expressed relative to controls as determined by ANOVA (adjusted *P*<0.01) in response to algal treatments. Bars within each column represent fold change difference between treatment and control. Red bars indicate up-regulation relative to control, green indicates down-regulation and black indicates no difference. All genes included in this analysis are in S2 Table.

**Figure 3:**
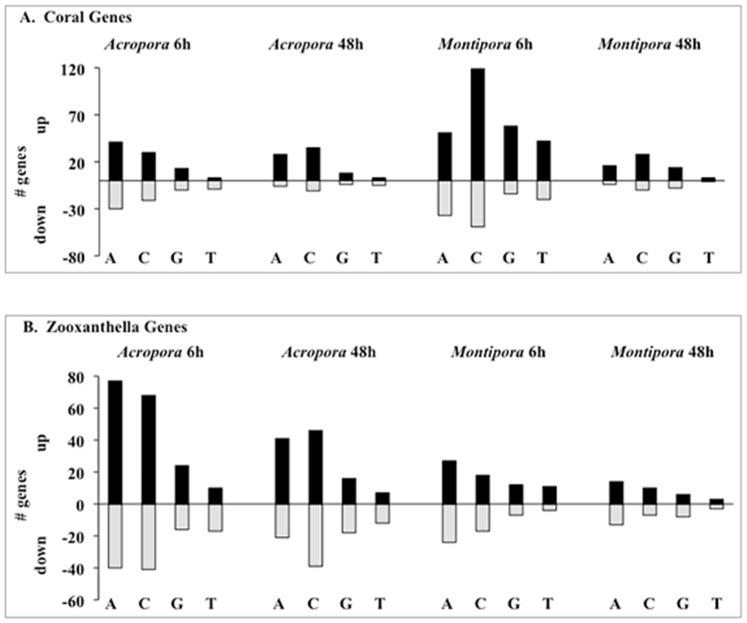
Number of differentially expressed genes (DEGs) in *Acropora millepora* and *Montipora digitata* coral (A) and *Symbiodinium* (B) genes for all treatments. Black bars indicate significantly up-regulated genes, and gray bars are significantly down-regulated genes relative to controls (adjusted *P* = 0.01). A =  *Amphiroa crassa*, C =  *Chlorodesmis fastigiata*, G =  *Galaxaura filamentosa* and T =  *Turbinaria conoides*.

Across all coral-algal interactions, 850 genes (441 coral and 409 *Symbiodinium*) were significantly differentially expressed relative to controls (Fig. 2; S2 Table; S3 Table). The number of differentially expressed genes (DEGs) varied between coral species, among macroalgal treatments, and over time for both coral (Fig. 3A) and *Symbiodinium* genes (Fig. 3B; S3 Table). 6 h exposures across all coral and macroalgal treatments produced higher mean expression levels than did 48 h exposures (0.246±0.022 and 0.065±0.015, respectively; t-test, *P* <0.0001).

**Figure 4:**
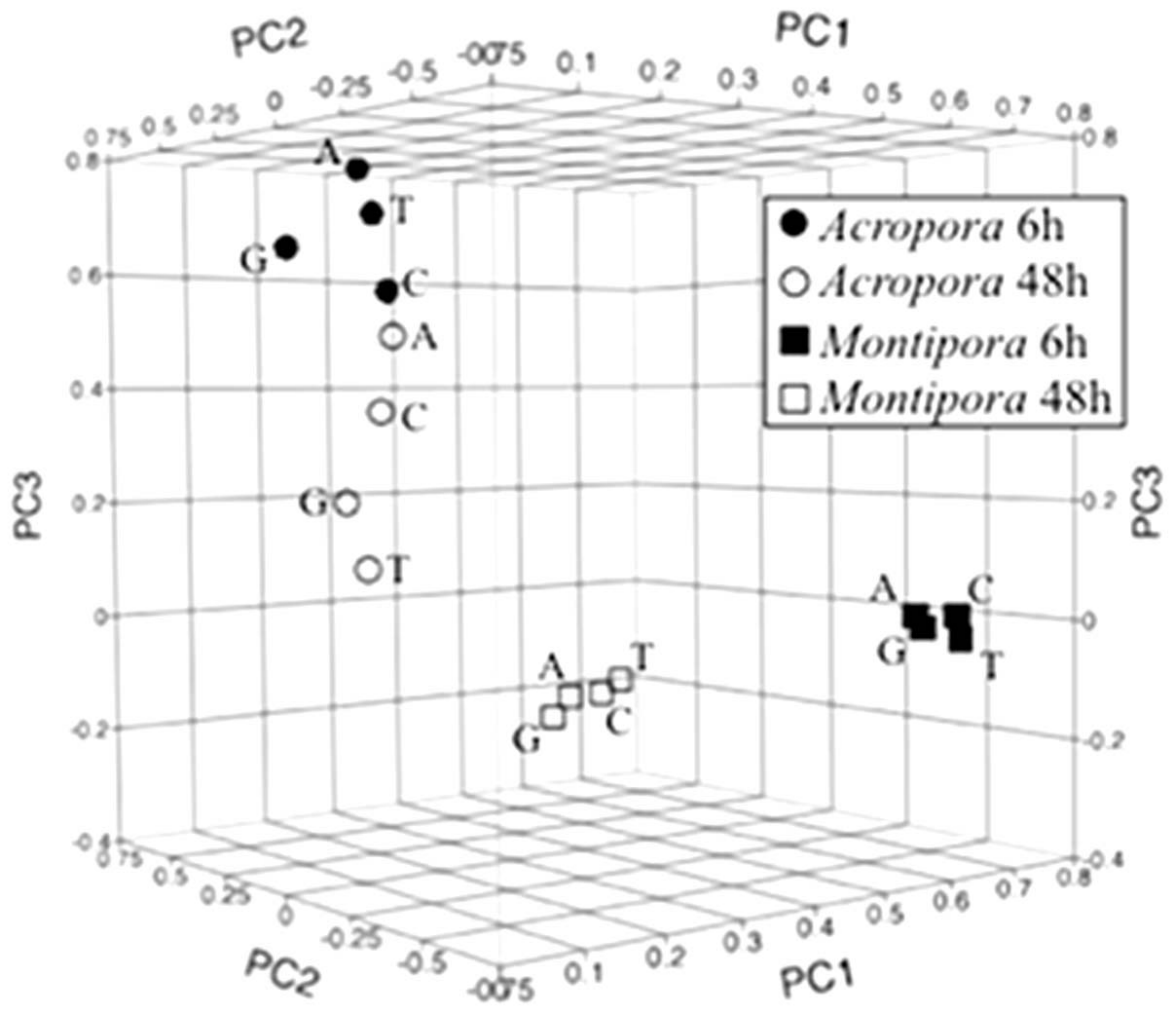
Multivariate grouping of coral exposure treatments using principle components analysis based on 850 differentially expressed *Acropora millepora* and *Montipora digitata* genes (both coral and *Symbiodinium*) relative to controls. All genes included in this analysis are in S2 Table. A =  *Amphiroa crassa*, C =  *Chlorodesmis fastigiata*, G =  *Galaxaura filamentosa* and T =  *Turbinaria conoides*.

To identify patterns of similarity across all experimental variables (coral species, macroalgal species, and duration of tissue exposure), principle component analysis (PCA) of expression intensities of the 850 DEGs revealed four non-overlapping clusters, with PC1, PC2 and PC3 explaining 55.0% of the variance (Fig. 4). PCA indicated that responses of *A. millepora* (coral and *Symbiodinium* genes combined) to all macroalgal species were more similar to each other at 6 h and 48 h along PC1 and PC2 than to responses of the *M. digitata* holobionts; however, macroalgal species separated along PC3. Within *M. digitata*, separation of clusters along PC2 corresponded to expression responses due to exposure time (6 h versus 48 h); whereas macroalgal species within both *M. digitata* groups were clustered.

### Coral Species Analysis

Mean expression levels (in terms of fold change relative to controls) of all DEGs were significantly less in *A. millepora* (all macroalgal treatments combined) compared to *M. digitata* (0.056±0.018 and 0.284±0.019, respectively; t-test, *P*<0.0001). Of the 177 *A. millepora* and 289 *M, digitata* coral genes differentially expressed (all macroalgal treatments combined), 25 were shared between corals, while 47 *Symbiodinium* DEGs were shared between the 323 *A. millepora* and 133 *M. digitata* DEGs (S2 Table). The direction of differential expression (up- or down-regulation relative to controls), however, was not always consistent between coral species (S2 Table). *A. millepora* and *M. digitata* coral and *Symbiodinium* exhibited some similar significant alterations in biological processes (protein binding, responses to unfolded proteins, cell differentiation activity, oxidoreductase activity and signal transduction) as a result of contact with macroalgae based on GO enrichment analysis of DEGs (Fisher's exact test; *P* = 0.01; Table 1A).

**Table 1:** Significantly over- or under-represented (Fisher's exact test; *P* = 0.01) biological processes and molecular functions of differentially expressed genes (DEGs) shared between coral species (A) and those specific to each coral (B and C).

GO-ID	Biological Process/Molecular Function	# of Genes	Adjusted *P*-Value	
**Shared ** ***Acropora*** ** and ** ***Montipora*** ** Coral Genes**				
GO: 0030235	nitric-oxide synthase regulator activity	2	0.009	over
GO: 0045597	positive regulation of cell differentiation	3	0.007	over
GO: 0006986	response to unfolded protein	3	0.002	over
GO: 0043021	ribonucleoprotein complex binding	2	0.009	over
GO: 0030911	TPR domain binding	2	0.009	over
GO: 0051082	unfolded protein binding	3	0.008	over
**Shared ** ***Acropora*** ** and ** ***Montipora*** ** Zooxanthella Genes**				
GO: 0005524	ATP binding	7	0.009	over
GO: 0009409	response to cold	2	0.006	over
***Acropora*** **-Only Coral Genes**				
GO: 0044260	cellular macromolecule metabolic process	38	0.000	under
GO: 0016705	oxidoreductase activity, acting on paired donors, with incorporation or reduction of molecular oxygen	10	0.001	over
GO: 0005543	phospholipid binding	11	0.003	over
GO: 0016776	phosphotransferase activity, phosphate group as acceptor	5	0.004	over
***Acropora*** **-Only Zooxanthella Genes**				
GO: 0045454	cell redox homeostasis	4	0.009	over
GO: 0009791	post-embryonic development	4	0.009	over
***Montipora*** **-Only Coral Genes**				
GO: 0003677	DNA binding	36	0.007	over
GO: 0006261	DNA-dependent DNA replication	8	0.006	over
GO: 0009880	embryonic pattern specification	4	0.009	over
GO: 0006897	endocytosis	9	0.001	over
GO: 0008285	negative regulation of cell proliferation	13	0.004	over
GO: 0004620	phospholipase activity	4	0.009	over
GO: 0010638	positive regulation of organelle organization	8	0.004	over
GO: 0006468	protein phosphorylation	40	0.002	over
GO: 0004674	protein serine/threonine kinase activity	31	0.000	over
GO: 0051101	regulation of DNA binding	4	0.009	over
GO: 0006275	regulation of DNA replication	8	0.004	over
GO: 2000602	regulation of interphase of mitotic cell cycle	9	0.004	over
***Montipora*** **-Only Zooxanthella Genes**				
None				

GO-ID indicates the identifier associated with the gene ontology term as defined by the Gene Ontology project (www.geneontology.org). Underlined adjusted *P*-value indicates under-represented gene ontology categories.

To understand species-specific gene expression responses of corals to macroalgal treatments, DEGs unique to each coral species were analyzed separately. After 6 h and 48 h exposures, more *A. millepora Symbiodinium* genes (293 and 200, respectively; all macroalgal treatments combined) were differentially expressed relative to coral genes (154 and 100, respectively; Fig. 3). The opposite pattern was observed in *M. digitata* where more coral genes (390 and 84, respectively) were differentially expressed after 6 h and 48 h exposures relative to *Symbiodinium* genes (120 and 64, respectively). Biological pathways uniquely affected in *A. millepora* coral were involved in transferase activity, phospholipid binding, and additional oxidation-reduction processes; however, there was a significant under-representation of genes involved in cellular macromolecule metabolic processes, while *Symbiodinium* exhibited alterations in redox homeostasis and developmental processes (Table 1B). Processes uniquely over-represented in *M. digitata* coral included DNA replication, protein modification, and RNA biosynthesis (Table 1C).

### Differential Response Due to Macroalgal Species

**Figure 5:**
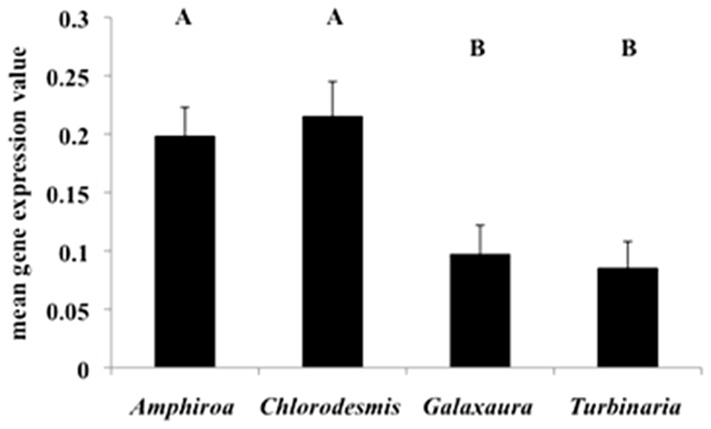
Mean gene expression (spot intensity) of all differentially expressed coral and *Symbiodinium* genes (combined) grouped by macroalgal treatment (ANOVA; *P* = 0.0003). Letters indicate significant groupings by Tukey-Kramer HSD post-hoc test.

**Figure 6:**
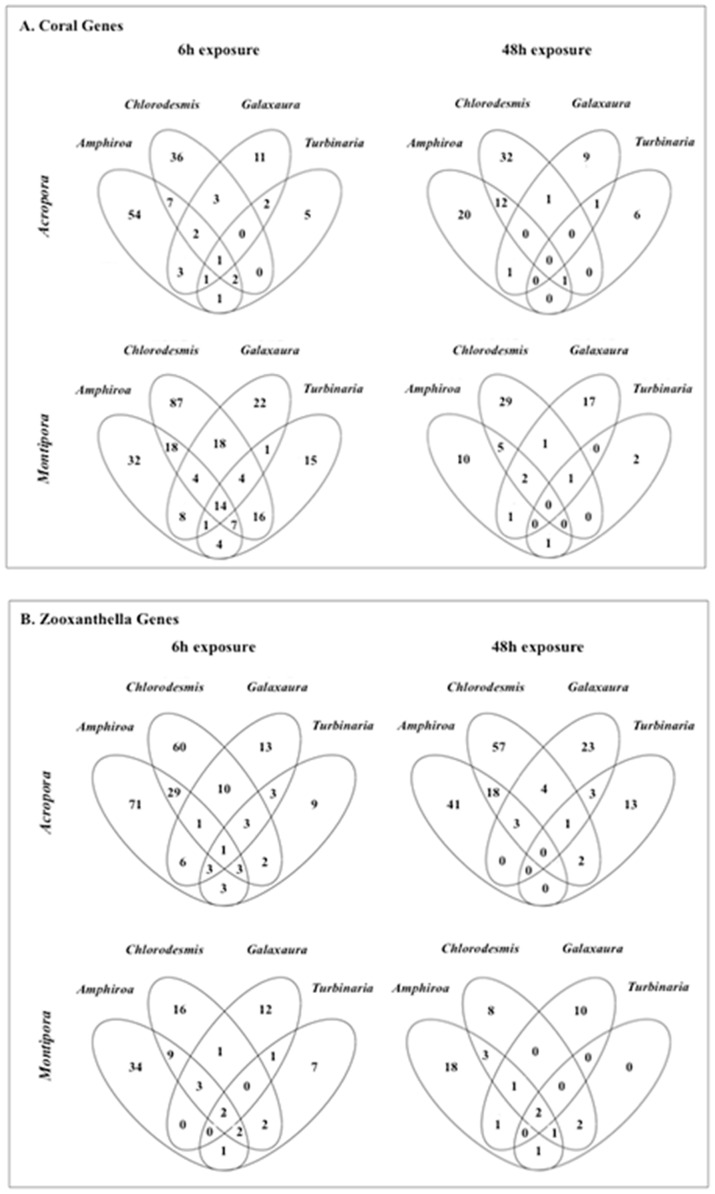
Overlaps of the Venn diagram showing the number of differentially expressed coral (A) and *Symbiodinium* (B) genes shared between and among macroalgal treatments within a coral species.

*A. millepora* and *M. digitata* coral and associated *Symbiodinium* displayed diverse alterations in gene expression in response to different macroalgal species (S5 Table). Exposure to *Amphiroa crassa* and *C. fastigiata* elicited significantly higher levels of gene expression (both coral species combined) than did exposure to *Galaxaura filamentosa* and *Turbinaria conoides* (Fig. 5; ANOVA, *P* = 0.0003). *A. crassa* and *C. fastigiata* treatments also shared the greatest number of affected genes across all treatments (19.1±4.6); however, most genes were differentially expressed in response to specific macroalgal species (Fig. 6), and not indicative of a general response to all macroalgae (e.g., of the 71 DEGs in *Acropora* following 6 h of contact with *Amphiroa*, 76% were unique to contact with *Amphiroa* as opposed to the other macroalgae (Fig. 6 upper left).

## Discussion

It is becoming increasingly clear that coral-macroalgal competition is complex, and commonly affected by macroalgal chemical traits instead of just physical effects of shading and abrasion [3], [4], [6], [10]–[12], [15], [17], [19], [22]–[26]. Reef ecologists thus need to appreciate not only the physical traits of macroalgae as competitors for light and substrate, but also their chemical traits, and the effects of these traits on the coral host, the host's mutualistic *Symbiodinium,* and the mutualistic and pathogenic microbes present in the holobiont's microbiome. Macroalgae are not just abrasives and shades, they are also localized chemical plants, some of which produce coral toxins [10], [28]. The rapid and variable molecular responses to different macroalgae that we documented here further emphasized this emerging, but incompletely formed, understanding.

Previous experiments [10] demonstrated that *Montipora digitata* was less impacted by macroalgal allelopathy than was *Acropora millepora*. Impact of competition was measured as mortality, bleaching, and photosynthetic suppression following contact with various macroalgal competitors (20d exposure) and their hydrophobic extracts (24 h exposure), including the four macroalgal species used in the present study. In the previous study, *M. digitata* was more resistant to contact with *Amphiroa crassa* and *Galaxaura filamentosa* relative to *A. millepora*, while both corals were unaffected by contact with *Turbinaria conoides* and both experienced bleaching and suppressed photosynthesis when in contact with *Chlorodesmis fastigiata*.

In conjunction with that study, gene expression analyses were conducted to characterize changes in transcriptional regulation of *A. millepora* in response to 20d contact with three of these macroalgae (*C. fastigiata, G. filamentosa* and *T. conoides*) [26]. Patterns of gene expression responses in that study were consistent with an imbalance between cellular oxidant species production and antioxidant capabilities of the coral holobiont. The greatest alteration in gene expression was observed after contact with *T. conoides*, the macroalgal species that had no effect on bleaching or photosynthetic activity. It was hypothesized that the significant transcriptional changes of *A. millepora*, including activation of reactive oxygen species (ROS)-induced apoptosis, in response to *T. conoides* enabled the coral holobiont to compensate for damage due to algal contact. *A. millepora* exhibited a reduced capacity to transcriptionally compensate for damage inflicted by *C. fastigiata* and *G. filamentosa*.

By investigating the changes in gene expression after shorter periods of contact (6 h and 48 h) in the present study, we hoped to determine whether confamilial corals respond to the same macroalgal competitor through similar early changes in gene expression despite differences in outcomes over long-term (20d) exposures, or if coral species differed in the biological pathways affected by the same macroalgae. *M. digitata*, which was more resistant than *A. millepora* to allelopathy from some macroalgae [10], demonstrated significant alterations in DNA binding, protein modification, protein serine/threonine kinase activity, and cell cycle and proliferation regulation relative to *A. millepora* (Table 1). These processes play a role in apoptosis, which can be caused by cellular stress and damage from macroalgal allelochemicals [26]. The early protective action of *M. digitata* in response to stress (<48 h of exposure) may be related to its greater resistance to some allelopathic macroalgae.

In addition to the inherent differences in responses of the corals themselves, differences in bleaching and transcriptional responses also may be influenced by different responses of *Symbiodinium* to the macroalgal competitors. Differences in physiological tolerances of associated *Symbiodinium* can directly affect the fitness of the host [34], [35]. *M. digitata* is less susceptible to thermally-induced bleaching [29] and damage by macroalgae than is *A. millepora* [10]. This may be related to the physiological tolerances of specific *Symbiodinium* types associated with these species (clade C or D in *A. millepora* and C in *M. digitata*, but a different lineage than in *A. millepora*) [29], [36]. These differences in symbiotic partners may influence the species-specific responses of corals to macroalgal contact observed in this study. Although we did not directly measure ROS in this study, gene expression results were consistent with elevated ROS in *A. millepora* after 6 h and 48 h exposure to some macroalgal species, with many redox processes significantly over-represented across multiple macroalgal exposure treatments (Table 2; S5 Table). These patterns in gene expression are also consistent with our previous study, from which we hypothesized that *A. millepora* transcriptionally responded to increased ROS due to damage from macroalgal contact after 20d of exposure [26].

**Table 2:** Cellular processes of corals commonly affected by external stressors.

	*Acropora*							
	Amphiroa*		Chlorodesmis*		Galaxaura*		*Turbinaria*	
	6 h	48 h	6 h	48 h	6 h	48 h	6 h	48 h
**apoptosis**					•			
**immune response**			•	•			•	
**oxidation-reduction process**	•	•		•	•	•		
**protein modification/transport**	•							
**ion homeostasis**		•			•	•		
**Ca^2+^ homeostasis**								
**cell signaling**				•	•	•		
**transcriptional regulation**								
**cytoskeletal reorganization**							•	

	*Montipora*							
	*Amphiroa*		Chlorodesmis*		Galaxaura*		*Turbinaria*	
	6 h	48 h	6 h	48 h	6 h	48 h	6 h	48 h
**apoptosis**			•	•			•	
**immune response**	•						•	
**oxidation-reduction process**		•	•					
**protein modification/transport**	•	•	•		•		•	•
**ion homeostasis**			•	•		•	•	
**Ca^2+^ homeostasis**						•		
**cell signaling**			•	•		•	•	
**transcriptional regulation**			•	•			•	
**cytoskeletal reorganization**							•	

Dots indicate an over-representation of biological processes and molecular functions of differentially expressed genes within these general response categories for each macroalgal treatment. Asterisks denote macroalgae that caused physiological damage (bleaching, mortality and reductions in photosynthetic activity of *Symbiodinium*) to the coral holobiont after 20d of contact [10].

There was, however, little transcriptomic evidence for alteration of processes involved in redox homoeostasis in *M. digitata*. In fact, there was a significant under-representation of oxidation-reduction processes represented in *M. digitata* in contact with *C. fastigiata* after 6 h. “Under-representation” indicates differential expression was observed in significantly fewer genes than predicted by chance based on the genes represented on our array as determined during the enrichment analysis (Fisher's Exact Test) in Blast2GO. Different *Symbiodinium* types exhibit distinct stress responses [32]. It is possible that symbiont differences between these two corals contributed to the divergence in transcriptomic responses between these species, and potentially to the greater resilience of *M. digitata*. Consistent with this possibility, macroalgal contact caused less than half as many DEGs for the *Symbiodinium* associated with *Montipora* as for those associated with *Acropora* (Fig. 6B).

From previous field experiments [10] and our previous gene expression study [26], we know that coral species exhibit different responses to various macroalgal competitors after 20d of contact. In the present study, we were interested in whether different macroalgae elicited similar or macroalgal-specific early stress responses in *M. digitata* versus *A. millepora*, and how gene expression patterns changed over time (6 h versus 48 h). *C. fastigiata* was the only macroalgae that suppressed photosynthetic activity in both coral species after 6 h of exposure; at 48 h, suppression was still apparent for *A. millepora*, but not *M. digitata* (Fig. 1), indicating rapid, but potentially short-term, differences in damage after contact with some macroalgal species. Not only do the allelopathic effects of *C. fastigiata* result in significant damage to these corals after long-term exposure (20d), but periods of contact as short as 6 h can produce significant reductions in photosynthetic activity.

To evaluate patterns of gene expression across coral species as a function of exposure time and macroalgal treatments, expression data of coral and *Symbiodinium* genes were interpreted at two levels: 1) as an overview of intensity and direction of expression of all DEGs relative to controls (up- or down-regulated) across all treatments, and 2) the number and identity of DEGs only in treatments in which the DEG was significantly different from the control. Hierarchical clustering analysis and PCA are methods to statistically group similar patterns of intensity and direction of expression of all DEGs identified in the gene expression analysis. Both methods grouped expression patterns (coral and *Symbiodinium* genes combined) by coral species and exposure time (Fig. 2 and 4), with differences between exposure times within coral species (e.g. *Montipora* 6 h versus *Montipora* 48 h). Overall expression patterns did not reveal similar responses of corals as a function of macroalgal species, otherwise we would have expected clustering by macroalgal treatment. Thus, when the intensity and direction of expression of all DEGs in all treatments were considered, coral species and exposure time accounted for the similarity in expression patterns.

When considering only the number and identity of the DEGs within the treatments in which it was significant (adjusted *P* = 0.01; expression data for non-significant treatments were not included in the analysis), the number of DEGs varied within coral/exposure time treatment (Fig. 3), and few DEGs were shared among macroalgal treatments within a coral and exposure time (Fig. 6). Pooling across all 32 contrasts, 56% of all DEGs were unique to a specific macroalga and only 1% responded to all 4 macroalgae (Fig. 6), indicating corals exhibited specific responses to different macroalgal species, and that responses were dynamic and varied over time.

These results were consistent with the hierarchical clustering analysis and PCA. Because there was no similar pattern of expression related to macroalgal species based on DEG number and identity (genes shared among treatments within a macroalgal species  = 1.4±0.4 and 1.8±0.6, coral and *Symbiodinium* genes respectively; data not shown), there was no consistent relationship of expression patterns defined by macroalgal species. When considering the total evidence (expression intensity and direction of all DEGs in all treatments regardless of their statistical significance relative to controls), genes generally showed similar trends in intensity and direction of expression within a coral/time exposure treatment, but the genes that were significantly different relative to controls were often unique across macroalgal treatments.

Although contact with *A. crassa* and *C. fastigiata* generally resulted in more DEGs (Fig. 3) and significantly greater overall expression levels (Fig. 5) in coral holobionts, only *C. fastigiata* significantly affected photosynthetic activity in this study (Fig. 1). We know from previous work that *C. fastigiata* allelopathy is more potent than *A. crassa* allelopathy, affecting all four coral genera it has been tested against, while *A. crassa* affects only two of the four and has a lower magnitude of effect in the two corals it impacts [9], [10]. This demonstrates that the identity and functions of affected genes were more important than the number of DEGs in determining the physiological outcome of the coral-macroalgal interactions. This also suggests that the molecular responses to *A. crassa* may have been effective in increasing the resistance of coral holobionts to this challenge, while a similar level of response to C. fastigiata was ineffective for *A. millepora*. However, we do not yet know which specific genes or biological pathways are critical in the health of the coral or maintenance of the coral/*Symbiodinium* symbiosis, nor do we fully know the specific macroalgal traits responsible for this damage [10],[26].

One pattern that emerged in this study was that coral-macroalgal pairings where coral holobionts were not damaged after 20d of exposure (*A. millepora*/*T. conoides*, *M. digitata/A. crassa* and *M. digitata/T. conoides*) [10] exhibited a significant early immune response after 6 h (Table 2; S4 and S5 Tables). Corals damaged after 20d of exposure to specific macroalgae generally did not exhibit significant changes in immune processes, with the exception of *A. millepora*/*C. fastigiata* interactions at 6 h and 48 h of exposure. The innate immune system of corals is more complex than once thought, and functions in defense against disease, as well as in symbiont recognition and acquisition [37], [38]. These immune responses may indicate activation of protective mechanisms against stressors associated with contact with some macroalgal competitors. Significant expression of immunity-related genes also may be linked to processes involved in maintaining the coral/*Symbiodinium* symbiosis in response to macroalgal contact. Because the mechanisms of bleaching are not fully understood, it is unclear what role these genes and biological pathways play during a stress event. We must further consider how the coral's microbiome is affected following macroalgal contact and whether early regulation of the innate immune system in corals that are more resistant to negative effects of contact suggests that indirect effects on microbes could be playing a role. There is clear evidence that some macroalgae produce metabolites that damage corals following contact [9], [10], [28], but whether this is direct or mediated indirectly by alterations in the coral's microbiome is unclear [11], [22]–[25].

A scleractinian cysteine-rich peptide gene (SCRiP 5), one of a group of scleractinian-specific genes [39] down-regulated during thermal stress [40], [41], was down-regulated in *A. millepora* in this study after 6 h exposure to *A. crassa*. In a previous study [26], a different SCRiP gene (SCRiP 6) was up-regulated in *A. millepora* in response to a 24 h exposure to hydrophobic extracts of *C. fastigiata*. Two other SCRiP genes (SCRiP 1, and 3a) were not differentially expressed in these studies. These transcriptional changes suggest that the function of some SCRiPs is not limited to temperature-induced stress responses.

Although many aspects of cellular stress response are evolutionarily conserved and often a general response to stress rather than stressor-specific [33], this study revealed divergence in transcriptional responses between two confamilial corals, as well as a diversity of dynamic responses within each coral species with respect to the species of macroalgal competitor and the duration of exposure to that competitor. Many biological processes in both the coral and *Symbiodinium* partners were significantly affected by contact with macroalgae, with the greatest responses observed after 6 h of contact and diminishing effects by 48 h. The coral-macroalgal interaction is complex and species-specific, as evidenced by the inherent differences in responses by coral hosts and their symbionts. These temporally complex and species-specific responses suggest that macroalgae are not simply suppressing corals via a uniform physical or chemical mechanism (e.g., shading, abrasion, or release of uniform primary metabolites), but are impacting corals via traits, and possibly mechanisms, that are unique to the coral-macroalgal pairings. The diverse responses of coral holobionts to different macroalgae that we document here may enable some corals to tolerate increases in macroalgal competitors. A more complete understanding of the stresses and responses involved may emerge as we develop a more robust understanding of the functions of the DEGs that we noted responding in this investigation.

## Methods

### Coral-algal Contact Experiment

Our coral-algal contact experiment exposed *Acropora millepora* and *Montipora digitata* fragments to one of four species of common macroalgae (*Amphiroa crassa*, *Chlorodesmis fastigiata*, *Galaxaura filamentosa,* and *Turbinaria conoides*) or an algal mimic made from Dacron line (White River Fly Shop), which served as a control for contact and shading. The algal mimic itself had no effect on coral bleaching, mortality or on photosystem II quantum yield of *Symbiodinium* [10], nor was there any significant effect on gene expression relative to corals with no algal mimic [26], so we rarely report data for this treatment.

Experimental coral fragments (6–8 cm height) were mounted to cement cones, held at a depth of 1 m (low tide), on steel racks on the reef flat at Votua Reef, Viti Levu, Fiji (18°13.0490 S, 177°42.9680 E), and allowed to acclimate for six weeks prior to the start of the experiment. Algal individuals of sizes representative of individuals in the local habitat were inserted between strands of 3-strand rope, and attached 1–2 cm from coral fragments (n = 10 for each algal species), allowing for algal movement and contact with the coral similar to that occurring in the field. To control for effects of the rope or other environmental effects that could erroneously be attributed to treatments, inert macroalgal mimics constructed from aggregations of Dacron line were similarly inserted between strands of rope and allowed to contact coral fragments (n = 10 for controls). The experiment was conducted within caged metal racks that excluded large herbivores and prevented their removal of treatment algae. Corals were sampled following 6 h or 48 h of exposure to macroalgae or mimic controls.

Investigations were undertaken following approval from the Fiji Ministry of Education, National and Heritage, Culture and Arts, Youth and Sports as well as the Korolevu-i-wai district elders and village environmental committees. Preserved coral samples were exported from Fiji with permission by the Fijian Ministry of Environment, and a CITES permit issued by the Fijian Islands CITES Management Authority. The US Fish and Wildlife Service approved import of these samples into the United States.

### Photosystem II Quantification

To determine physiological effects, in terms of changes in photosynthetic activity, of *in hospite Symbiodinium* of *A. millepora* and *M. digitata*, after exposure to each macroalga, pulse amplitude modulated (PAM) fluorometry (Diving-PAM, Walz) was used to quantify effective quantum yield of photosystem II (Φ_PSII_). This measurement was used as a proxy for bleaching because *Symbiodinium* density measurements could not be made on the same tissue used in the genetic expression analysis. Light-adapted measurements of Φ_PSII_ were taken at the point of coral-algal contact for 8–10 coral fragments per treatment. For controls, n = 8–10 for each coral species for each exposure duration (6 h and 48 h), measurements were taken from similar positions on the coral fragment where the coral was in contact with the artificial alga. Measurements were taken 4–5 mm from, and perpendicular to, the coral surface while avoiding self-shading. All measurements were taken between 1230–1500 h, and measures of different treatments were interspersed to prevent confounding treatment effects with effects of temporal changes in environmental conditions.

### RNA Preparation, Microarray Design and Analysis

Immediately after recording PAM readings, the coral portion that had been in contact with each macroalga or algal mimic was preserved in Trizol (Invitrogen) and frozen. Coral tissue was scraped from the calcium carbonate skeleton where algal contact had occurred and total RNA was extracted according to the manufacturer's protocol. Total RNA was purified using RNeasy MinElute Clean-up kit (Qiagen), and RNA pellets were resuspended in nuclease-free water. RNA concentration and quality were assessed using a NanoDrop ND-1000 spectrophotometer (ThermoScientific). RNA integrity was confirmed using the RNA 6000 Nano kit and Bioanalyzer 2100 (Agilent Technologies).

RNA (5 ng) from 3–7 biological replicates for each algal exposure treatment and control, as well as RNA from technical replicates (n = 6) verifying consistency among arrays, was processed and labeled using the One-Color Microarray-Based Gene Expression Analysis kit (Agilent Technologies). Data from technical replicates was not included in the final analysis. Microarray hybridization followed manufacturer's protocol.

A custom microarray that included 1,029 and 853 unique coral and *Symbiodinium* genes, respectively, representing a range of functional pathways, was designed and used to measure changes in gene expression as a result of contact with macroalgae. Genes were acquired from bioinformatic mining of recent transcriptome sequencing projects (www.medinalab.org/zoox/ [42]) and submissions into public databases (GenBank, www.ncbi.nlm.nih.gov/genbank/). At the time of array design, there were few *M. digitata* sequences publically available to use during the probe design process. Open reading frames from candidate anthozoan genes were blasted in GenBank to determine probable gene identity with an E-value cutoff of e^−6^ and to determine that these gene regions were conserved across phylogenetically diverse taxa (typically non-cnidarian invertebrates for coral genes and apicomplexan or plant genes for *Symbiodinium*). Few genes were restricted to only scleractinian coral species (e.g. scleractinian cysteine-rich peptide genes). Two 60-mer probes for each gene were designed from open reading frames using eArray (Agilent Technologies), and replicated 3–4 times on the microarray in addition to positive (spike-in) and negative controls. A total of 1,185 and 1,061 coral and *Symbiodinium* genes with some replicated genes with sequences from different species were spotted on the array (S1 Table). To avoid overrepresentation of single genes in the analysis, expression data from only one homologous gene (the first listed in the probe report) were included in the analysis (direction of expression were the same for the homologous genes, but intensities varied; data not shown). Arrays were scanned using Agilent G2505C Microarray Scanner and Feature Extraction Software 10.7.1.1 (Morehouse School of Medicine, Atlanta, GA). Data were statistically analyzed using JMP Genomics 3 (SAS Institute).

Raw spot intensity data were log2 transformed and loess normalized. Background intensity was subtracted from each feature and replicate probes for each gene were averaged. Analysis of variance (ANOVA) was used to detect highly significant expression differences between control, and treatment corals for all pairwise comparisons (*P*<0.01). By identifying significant differences in gene expression of macroalgal treatments relative to corresponding controls, and not treatment by treatment comparisons, DEGs represented effects from specific treatments and not due to species-specific artifacts (e.g. nucleotide sequence difference between species) unrelated to treatments, or diel cycles of gene expression [43]. Significance levels were adjusted using a false discovery rate correction to control for multiple testing [44]. Hierarchical cluster analysis of significant genes using Ward's method was performed between coral species, macroalgal species and exposure times resulting in clusters based on similarities in gene expression patterns. Principle component analysis (PCA) was conducted to identify patterns of similarity across all experimental variables (coral species, macroalgal type, and exposure time). Analyzing significant gene ontology categories rather than individual genes increases the confidence that a specific biological process is involved in the response to a stimulus since there is evidence that multiple genes are behaving in a consistent, functional manner; therefore, gene functions and ontologies (GO terms) were obtained from the European Bioinformatics Institute (EMBL-EBI) (www.ebi.ac.uk), UniProt (www.uniprot.org), and analyzed using Blast2Go v2.6.4 [45].

## Supporting Information

S1 Table Coral (A) and *Symbiodinium* (B) genes, including putative gene name, accession number and E-value, included on the coral holobiont microarray used in this study. Accession numbers are from GenBank and www.medinalab.org/zoox/ [41].
(XLS)

S2 Table Significant fold changes of *Acropora millepora* and *Montipora digitata* coral (A) and *Symbiodinium* (B) genes significantly (*P* = 0.01) altered by macroalgae after 6 h or 48 h of contact. Positive values indicate up-regulation of expression relative to controls, while negative values indicate down-regulated genes. A =  *Amphiroa crassa*, C =  *Chlorodesmis fastigiata*, G =  *Galaxaura filamentosa* and T =  *Turbinaria conoides*.
(XLS)

S3 Table Putative identification and fold change of differentially expressed coral (A) and *Symbiodinium* (B) genes shared between 6 h and 48 h treatments for each coral species/macroalgal comparison. Positive values indicate up-regulation of expression relative to controls, while negative values indicate down-regulated genes.
(XLS)

S4 Table Significantly over-represented (Fisher's exact test; *P* = 0.01) biological processes and molecular functions of differentially expressed coral (A) and *Symbiodinium* (B) genes (DEGs) shared between 6 h and 48 h treatments for each coral species/macroalgal comparison. GO-ID indicates the identifier associated with the gene ontology term as defined by the Gene Ontology project (www.geneontology.org).
(XLS)

S5 Table Significantly over- and under-represented (Fisher's exact test; *P* = 0.01) biological processes and molecular functions of differentially expressed coral (A) and *Symbiodinium* (B) genes (DEGs) *not* shared between 6 h and 48 h treatments for each coral species/macroalgal comparison. Underlined adjusted *P*-value indicates under-represented gene ontology categories.
(XLS)
